# Slicer-independent mechanism drives small-RNA strand separation during human RISC assembly

**DOI:** 10.1093/nar/gkv937

**Published:** 2015-09-17

**Authors:** June Hyun Park, Chanseok Shin

**Affiliations:** 1 Department of Agricultural Biotechnology, Seoul National University, Seoul 151-921, Republic of Korea; 2 Research Institute of Agriculture and Life Sciences, Seoul National University, Seoul 151-921, Republic of Korea; 3 Plant Genomics and Breeding Institute, Seoul National University, Seoul 151-921, Republic of Korea

## Abstract

Small RNA silencing is mediated by the effector RNA-induced silencing complex (RISC) that consists of an Argonaute protein (AGOs 1–4 in humans). A fundamental step during RISC assembly involves the separation of two strands of a small RNA duplex, whereby only the guide strand is retained to form the mature RISC, a process not well understood. Despite the widely accepted view that ‘slicer-dependent unwinding’ via passenger-strand cleavage is a prerequisite for the assembly of a highly complementary siRNA into the AGO2-RISC, here we show by careful re-examination that ‘slicer-independent unwinding’ plays a more significant role in human RISC maturation than previously appreciated, not only for a miRNA duplex, but, unexpectedly, for a highly complementary siRNA as well. We discovered that ‘slicer-dependency’ for the unwinding was affected primarily by certain parameters such as temperature and Mg^2+^. We further validate these observations in non-slicer AGOs (1, 3 and 4) that can be programmed with siRNAs at the physiological temperature of humans, suggesting that slicer-independent mechanism is likely a common feature of human AGOs. Our results now clearly explain why both miRNA and siRNA are found in all four human AGOs, which is in striking contrast to the strict small-RNA sorting system in *Drosophila*.

## INTRODUCTION

Small RNA-mediated silencing pathways are essential mechanisms that regulate gene expression in many eukaryotic organisms. Small RNAs function through a cytoplasmic ribonucleoprotein complex, known as the RNA-induced silencing complex (RISC), whose core component is a member of the Argonaute (AGO) subfamily of proteins, of which there are four in humans (AGOs 1–4) (1). The assembly of a small RNA duplex into the RISC mainly comprises two steps: RISC loading and strand separation (duplex unwinding) (2). Small RNA duplexes are actively incorporated into AGO proteins to form pre-RISCs, which requires the Hsc70–Hsp90 chaperone machinery (3–6). This machinery, which is fueled by ATP hydrolysis, drives a dynamic conformational change in the AGO proteins, thereby allowing them to accommodate the duplex (3–6). The two strands of the duplex are subsequently unwound within the AGO proteins, in which one of the strands (the guide strand) is retained, while the other strand (the passenger-strand) is discarded to form the mature RISC. The specific selection bias of the guide strand is governed by the relative thermodynamic asymmetry of the first few bases at each end of the duplex, as the strand with less stably paired 5′ end preferentially serves as the guide (7,8). Given that small RNAs are loaded into AGO proteins as double strands, the strand selection is already determined by the polarity of small RNA duplexes upon RISC loading prior to the duplex unwinding (2,9).

There are two types of duplex unwinding: slicer-dependent and -independent (2). Slicer-dependent unwinding relies on an AGO2-mediated slicer activity that cleaves the passenger-strand to facilitate its rapid removal (10–13). Therefore, the siRNA duplexes that are incorporated into slicer-deficient AGOs (1, 3 and 4) cannot be unwound by the slicer-assisted pathway. In contrast, most miRNA duplexes undergo slicer-independent unwinding because they often contain seed and/or central mismatches. Beyond the slicer-assisted pathway for highly complementary siRNAs, how slicer-independent unwinding occurs, under what conditions, and whether additional factors are needed, remains largely unknown (14).

The initial motivation for this study was provided by the fact that the thermodynamic properties of the guide-target duplex greatly affect the efficacy of target recognition and silencing (15–17). As previously proposed, slicer-independent unwinding can be viewed as the reverse process of target RNA recognition, with the passenger-strand being regarded as a target of the guide strand (2,18,19). Therefore, we hypothesized that the thermodynamic properties of the guide-passenger duplex may also play a role. Indirect support for this idea was provided by the observation that the thermostability of the stem structures of small hairpin RNAs greatly affects the loading of AGO proteins (20). These accumulating observations led us to carefully examine slicer-independent unwinding in human RISCs using various biochemical approaches. Of these, a native gel system provides a powerful tool for detecting RISC complexes assembled with small RNA duplexes (21,22) or precursor miRNAs (23) from mammalian cell lysates.

Here we carefully revisited the role of AGO2 slicing activity in RISC assembly, now showing that in human cells, the elevated physiological temperature dramatically contribute to strand separation, even when slicer-assisted pathway is absent. In contrast to the previous models, our in-depth biochemical analyses showed that cleavage-deficient AGOs can also be programmed with highly complementary siRNAs at the physiological temperature of humans. In addition, our functional analysis with several mutant AGO proteins supports the previous hypothesis that slicer-independent unwinding is mediated by the functional domains of the AGO protein (18,19,21,22) that experience dynamic conformational changes during its catalytic cycle (24–27). Altogether, our data reveal that the RISC maturation process has many more nuances, and that these events can be fine-tuned in various organisms according to their body temperature.

## MATERIALS AND METHODS

### Cell culture and siRNA transfection

HEK293T and HeLa S3 cells were cultured in Dulbecco's modiﬁed Eagle's medium supplemented with 10% (v/v) fetal bovine serum (FBS) in 5% CO_2_. Lipofectamine 2000 was used for siRNA transfections according to the manufacturer's protocol (Invitrogen). *Drosophila* S2 cells were cultured at 25°C in Schneider's medium supplemented with 10% FBS.

### Cell lysate preparation

HEK293T cells at 50% confluence were transfected with FLAG-tagged AGO expression plasmids (10 μg per 100-mm dish) using the calcium phosphate method and harvested after 48 h. Naïve HeLa S3 and *Drosophila* S2 cells were used for lysate preparation. Cells were washed three times with cold PBS, pH 7.4, and collected by centrifugation at 3,000 rpm at 4°C for 5 min. Cell pellets were resuspended in two packed-cell volumes of hypotonic lysis buffer (20 mM HEPES-KOH, pH 7.4, 10 mM KOAc, 1.5 mM Mg(OAc)_2_, 5 mM DTT, 0.1% Tween-20 and 1× EDTA-free Protease Inhibitor cocktail [Roche]) and incubated for 10 min on ice. Subsequently, the cytoplasmic fraction was clarified by centrifugation at 15,000 rpm at 4°C for 20 min. The supernatant was flash frozen in liquid nitrogen and immediately stored at –80°C in single-use aliquots. To obtain the expression plasmids encoding the FLAG-tagged human AGO proteins, the coding region of each cDNA fragment was inserted into pcDNA-based vectors (Invitrogen). Non-slicer (D597A), N domain (F181A) and truncation (ΔPAZ and ΔN) mutants of AGO2 were generated by site-directed mutagenesis.

### General methods for *in vitro* RNAi

*in vitro* RNAi reactions were performed under standard assay condition (28) that typically contained 5 μl of cell lysate, 3 μl of reaction mix (28), 1 μl of small RNA duplex and 1 μl of target substrate in a 10-μl reaction volume. The reaction temperature and final concentration of magnesium are indicated in the figures, and the details of each assay are described below. Phosphorimaging was performed using a BAS-2500 image analyzer (Fujifilm) and signal intensities were quantified using MultiGauge (Fujifilm). The sequences of the oligonucleotides used for the assays are shown in Supplementary Table S1.

### Target RNA cleavage assay

To prepare targets, DNA fragments containing the target site were amplified by PCR, *in vitro* transcribed and radiolabeled at the 5′-cap by guanylyl transferase and [α-^32^P] GTP (3,000 Ci/mmole, PerkinElmer) using the mScript mRNA production system (Epicentre) according to the manufacturer's instructions, followed by denaturing polyacrylamide gel purification. Fifty nanomolar of 5′-phosphorylated small RNA duplex was pre-incubated before the addition of ∼5 nM of ^32^P-cap-radiolabeled target RNA. The reactions were quenched by adding 2× proteinase K buffer (200 mM Tris–HCl, pH 7.5, 25 mM EDTA, 300 mM NaCl and 2% [w/v] SDS), 2 mg/ml proteinase K, and 1 μg glycogen, incubated for 15 min at 60°C. After ethanol precipitation, the target substrate (78-nt) and 5′ cleavage product (37-nt) were analyzed in a 10% denaturing polyacrylamide gel.

### *in vitro* RISC assembly assay

*in vitro* RISC assembly assays were performed essentially as described previously (29). Ten nanomolar of guide strand radiolabeled duplexes (i.e. a 5′-^32^P-radiolabeled guide strand annealed to an unlabeled phosphorylated passenger-strand) were incubated in the standard reaction mixture with 10 nM of 2′-*O*-methyl ASO as a target for native gel analysis. The RISC complexes were resolved by vertical native 1.4% agarose gel electrophoresis at 300V in a 4°C cold room.

### Passenger-strand cleavage assay

*in vitro* RNAi reactions were performed as described above, but used 10 nM of passenger-strand radiolabeled duplexes and 1 μM of 2′-*O*-methyl oligonucleotide complementary to the passenger-strand to protect the cleavage product from degradation. After proteinase K digestion and ethanol precipitation, the samples were analyzed in a 15% denaturing polyacrylamide gel.

### Immunopurification of AGO2 complex and unwinding assay

Ten nanomolar of guide strand radiolabeled duplexes were incubated in lysates from HEK293T cells expressing FLAG-hAGO2. Ten percent of the reaction was removed to serve as the input fraction and the rest of the sample was incubated with anti-FLAG M2 Affinity Gel (Sigma) for 2 h with gentle rocking at 4°C, followed by six washes with 10× bead-volumes of wash buffer containing 150 mM NaCl. RNA samples were then phenol extracted, ethanol precipitated and resolved by 15% native polyacrylamide gel electrophoresis at 4°C as described previously (30).

### *in vitro* deadenylation assay

For the preparation of poly(A)-tailed target RNA, DNA fragments containing the target site were amplified by PCR, *in vitro* transcribed and purified by UV shadowing on denaturing polyacrylamide gel. Subsequently, the purified substrate (A_0_) was poly-adenylated by poly(A) polymerase (Takara) and radiolabeled at the 5′-cap by guanylyl transferase and [α-^32^P] GTP (3,000 Ci/mmol, PerkinElmer) using the mScript mRNA production system (Epicentre) according to the manufacturer's instruction, followed by denaturing polyacrylamide gel purification. Radiolabeled A_0_ (without the poly(A) tail) substrate was used as the migration marker for the completely deadenylated fraction. Deadenylation by AGO1-RISC was performed with lysates from HEK293T cells expressing FLAG-AGO1. Fifty nanomolar of 5′-phosphorylated small RNA duplex was pre-incubated for 30 min before the addition of ∼5 nM of ^32^P-cap-radiolabeled poly(A)-tailed target RNA. After proteinase K digestion and ethanol precipitation, the samples were analyzed in a 6% denaturing polyacrylamide gel.

### Native gel mobility-shift assay

Ten nanomolar of radiolabeled ssRNAs or duplex RNA was incubated with increasing concentrations (0–200 nM) of purified recombinant hAGO2 in a 10-μl reaction containing 10 mM HEPES–KOH, pH 7.4, 50 mM KCl, 1 mM DTT, 3% (w/v) Ficoll-400 and 0.1 mg/ml BSA. RNA–protein complexes were analyzed by 6% native polyacrylamide gel electrophoresis at 120V in a 4°C cold room.

### Northern blotting

RNAs were resolved in a 15% denaturing polyacrylamide gel, electrophoretically transferred to Hybond-NX membrane (GE healthcare), and chemically cross-linked via 1-ethyl-3-(3-dimethylaminopropyl) carbodiimide; EDC method (31) (Sigma). The DNA probe was radiolabeled using T4 polynucleotide kinase (Takara) and [γ-^32^P] ATP (6,000 Ci/mmol, PerkinElmer) and hybridized with the membrane using PerfectHyb Plus (Sigma).

### Western blotting

The primary antibodies included polyclonal rabbit anti-FLAG (1:5,000; Sigma), polyclonal rabbit anti-beta tubulin (1:15,000; Abcam) and monoclonal mouse anti-GAPDH (1:10,000; Abcam). The secondary antibodies for chemiluminescent detection were horseradish peroxidase-conjugated goat anti-rabbit (or donkey anti-mouse) IgG antibodies (Jackson ImmunoResearch).

### Expression and tandem affinity purification of recombinant human AGO2

To obtain N-terminal FLAG-tagged protein, oligonucleotides corresponding to the FLAG peptide were inserted into the pFastBac-His plasmid (Invitrogen). Recombinant hAGO2 proteins were expressed in insect cells using the Bac-to-Bac Baculovirus Expression System (Invitrogen) according to the manufacturer's instructions. Briefly, recombinant baculovirus DNA was transfected using Cellfectin II Reagent (Invitrogen) into Sf9 cells grown at 26°C in Sf-900 III medium (Invitrogen). Sf9 cells were infected with the recombinant virus for 48 h, harvested and washed with PBS. Cells were lysed in lysis buffer (20 mM HEPES–NaOH, pH 7.4, 300 mM NaCl, 0.5% Triton X-100, 5% glycerol, 15 mM imidazole, 2 mM β-mercaptoethanol, and 1× EDTA-free Protease Inhibitor cocktail [Roche]) and sonicated five 6-s bursts at 35% amplitude. After centrifugation at 18,500 rpm at 4°C for 25 min, the supernatant was loaded onto a Ni-NTA affinity column (GE Healthcare), washed with an imidazole gradient (20–75 mM) and eluted in 250 mM imidazole. The recombinant protein was further purified via affinity binding to anti-FLAG M2 Affinity Gel (Sigma), eluted with a 3× FLAG peptide (Sigma) and stored in aliquots containing 10% (v/v) glycerol at –80°C.

## RESULTS

### Small RNA maturation is highly dependent on the ambient temperature both *in vitro* and in cells

**Figure 1.**
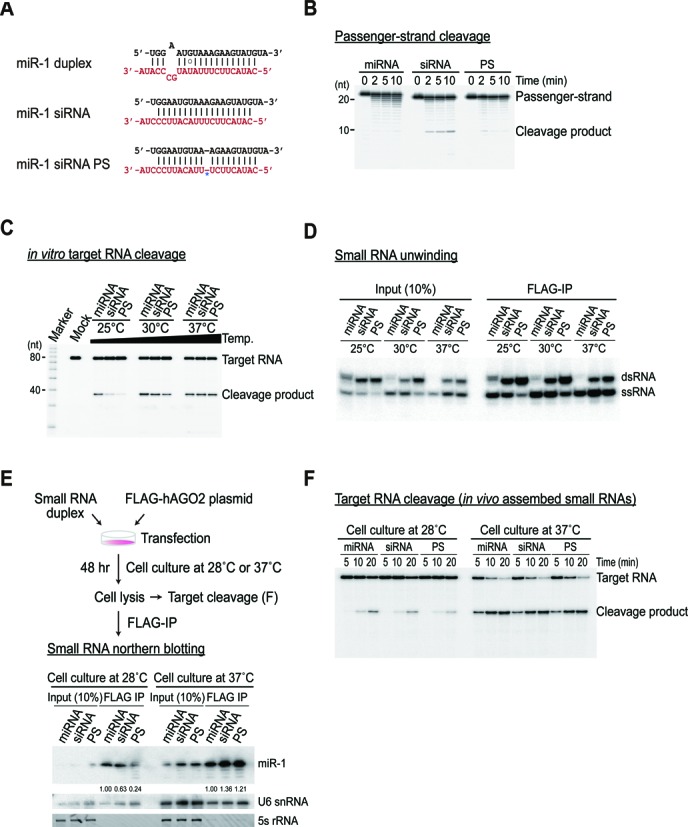
Small RNA maturation is highly sensitive to the ambient temperature both *in vitro* and *in vivo*. (**A**) Small RNA duplexes used in this study; miR-1 with an endogenous duplex structure, functionally asymmetric miR-1 siRNA (miR-1 was perfectly paired to its antisense, except for the first position from the 5′ end) and miR-1 siRNA PS (miR-1 siRNA introduced with phosphorothioate linkage at the scissile phosphate, denoted by a star). The guide and passenger-strands are shown in black and red, respectively. (**B**) PS modification inhibits the cleavage of the passenger-strand. Small RNA duplexes containing radiolabeled passenger-strands were incubated in lysates expressing tagged AGO2 for the indicated times at 25°C. (**C**) Distinct efficacy of target cleavage at different temperatures for each different small RNA duplex. Small RNAs duplexes were assembled in lysates expressing tagged AGO2 for 15 min at the indicated temperature (mock refers to the no duplex control). Cap-radiolabeled target RNA was then added and further incubated for 15 min at the indicated temperatures. (**D**) Distinct efficacy of unwinding at different temperatures for each different small RNA duplex. Small RNA duplexes carrying radiolabeled guide strands were incubated in lysates from expressing tagged AGO2 for 30 min at the indicated temperature. Small RNAs incorporated into the AGO2 complexes were immunopurified and analyzed in native gel, along with the 10% input (IN) relative to the IP. (**E**) Small RNA maturation is dependent on the ambient temperature in living cells. HEK293T cells were co-transfected with 10 nM of small RNA duplexes and FLAG-AGO2 plasmids. Cells were then cultured either at 28°C or 37°C and harvested at 48 h post-transfection; cell lysates were subjected to FLAG-IP, followed by northern blotting with the miR-1 probe. The blot was probed for U6 snRNA as a loading control. Ethidium bromide-stained 5S rRNA served as another loading control. The numbers below the blot are the relative expression levels, normalized using the U6 snRNA loading control. (**F**) Cell lysates prepared as in panel (E) were used for the target cleavage assay. Cap-radiolabeled target RNA was added and incubated for the indicated times.

We first established a cell-free system derived from human embryonic kidney (HEK) 293 cells expressing human AGO2 (hAGO2) (21,22,32). We and others (21,22) have found that a naïve HEK293T cell lysate on its own is not competent to reconstitute RNA interference (RNAi) *in vitro* (Supplementary Figure S1A and B), possibly because endogenous AGOs are largely pre-occupied by endogenous miRNAs (3,6,33,34). Transient expression of AGO2 generated free RISCs for exogenous RISC programming, which efficiently recapitulate RNAi *in vitro* (Supplementary Figure S1A–C). To specifically examine the relative contribution of slicer-dependent and -independent unwinding of AGO2, we initially used three model duplexes (Figure 1A): endogenous miR-1, miR-1 siRNA (functionally asymmetric) and miR-1 siRNA with a phosphorothioate (PS) modification of the scissile phosphodiester bond that inhibits passenger-strand cleavage (10) (Figure 1B). To exclude the possibility of endogenous miRNAs participating in RNAi, miR-1, which is specifically expressed in heart and skeletal muscle (35), was used, and its absence in HEK293T cells was confirmed by northern blotting (Supplementary Figure S1D).

We initially performed a classical *in vitro* target cleavage and unwinding assay at various temperatures to investigate the thermodynamic properties of small RNA maturation. The miRNA exhibited the most efficient target cleavage and unwinding at 25°C, followed by the siRNA and PS duplexes, which is generally consistent with previous findings in *Drosophila* (10,11,13) (Figure 1C and D). Unexpectedly, the defects of the siRNA and PS duplexes were largely rescued by increasing the temperature to 30°C (Figure 1C and D). At the physiological temperature (37°C) of human cells, all three duplexes exhibited a similar level of target cleavage (Figure 1C) and produced a comparable amount of AGO2-bound ssRNA in the immunoprecipitation (IP) fraction (Figure 1D). These results indicated that the ‘slicer-dependency’ of unwinding is largely influenced by the temperature. To examine whether this mechanism was operating *in vivo*, we co-transfected HEK293T cells with the FLAG-AGO2 plasmid and small RNAs. The cells were cultured at 28°C or 37°C, and the relative amount of small RNAs and their functional activities were measured by northern blotting and a target cleavage assay, respectively (Figure 1E and F). The results were mostly consistent with the *in vitro* findings that small RNA maturation is highly dependent on the ambient temperature, which also seemed to have a drastic effect on the degree of passenger-strand cleavage.

### Slicer-dependent unwinding increases with Mg^2+^ concentration and decreases with increasing temperature

**Figure 2.**
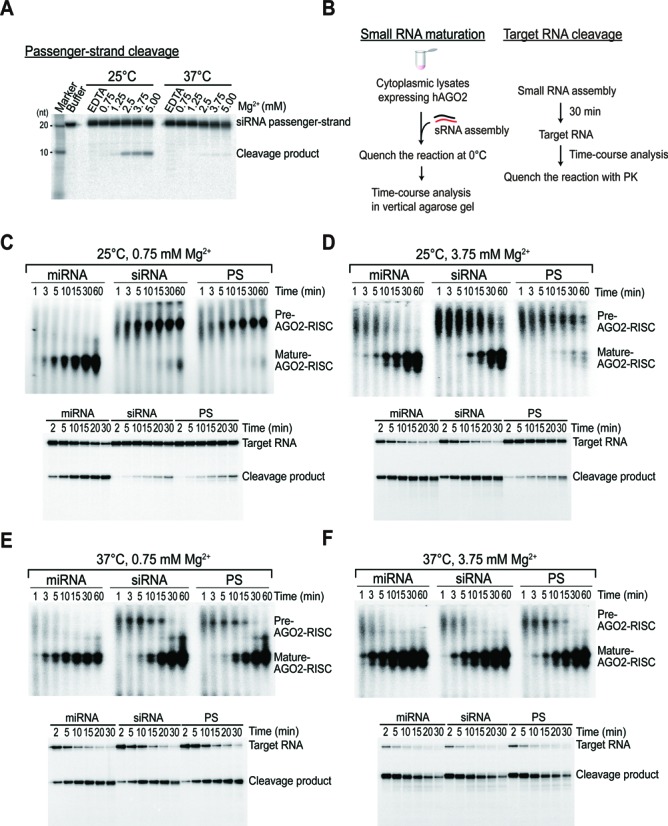
Slicer-dependency is determined by temperature and the Mg^2+^ level. (**A**) The effect of temperature and Mg^2+^ on the degree of passenger-strand cleavage. siRNA duplexes containing radiolabeled passenger-strands were incubated in lysates expressing tagged AGO2 for 15 min either at 25°C or 37°C, with Mg^2+^ concentrations ranging from 0 to 5 mM. The incubation with 5 mM EDTA served as a negative control. (**B**) A schematic of the analysis for (C–F). (**C**–**F**) The slicer-dependency of RISC assembly decreases with increasing temperature and decreasing Mg^2+^ levels. Small RNA duplexes containing radiolabeled guide strands were incubated in lysates expressing tagged AGO2 at the indicated temperature and Mg^2+^ concentrations for the indicated times. Under the same condition, target cleavages were analyzed after 30 min of small RNA assembly. Representative data of at least two independent experiments are shown.

Divalent cations, particularly Mg^2+^, have been shown to have a unique role in nucleic acid duplex stabilization (36,37). Given that cleavage-competent AGO proteins are also Mg^2+^-dependent endonucleases (38), we hypothesized that the extent of passenger-strand cleavage may be influenced by the Mg^2+^ concentration. To this end, we performed a passenger-strand cleavage assay using the siRNA duplex in which the Mg^2+^ concentration ranged from 0 to 5 mM. Cleavage was abrogated by the addition of EDTA, indicating that Mg^2+^-dependent AGO2-slicer activity is required (Figure 2A). Cleavage products increased significantly with Mg^2+^ levels at 25°C (Figure 2A). These results explained why the cleavage of not only the PS duplex, but also the siRNA, was not very efficient at 25°C in the presence of 1 mM Mg^2+^ (Figure 1C). In contrast, the cleavage products were barely detectable at 37°C, despite the presence of a 100-fold excess of a 2′-*O*-methylated trap (10,13) that protects them from degradation (Figure 2A). These results suggested that slicer-dependency decreased with increasing temperature, and it raised an intriguing question regarding how Mg^2+^, together with temperature, influences duplex unwinding during RISC assembly. Because RISC assembly is a transient process, it is fundamentally important to understand its kinetics at the early stage. To do so, we employed an *in vitro* reconstitution system using native agarose gels, which was developed by Tomari and his colleagues (19,21,22,29). While RISC complexes cannot be detected using naïve HEK293T cell lysates (21,22), the transient expression of each AGO protein enables the sensitive detection of the two complexes formed by exogenous RISCs, including pre- and mature RISCs (21) (Supplementary Figure S2A-C). Here, we exploited this reconstitution system to further biochemically dissect and functionally characterize duplex unwinding by human AGO proteins (Figure 2B).

### High temperature drives duplex unwinding largely independently of slicer-activity

We set out to explore how temperature and Mg^2+^ affect duplex unwinding during RISC assembly. At 25°C, only the miRNA showed an efficient conversion of pre- to mature-AGO2-RISC, regardless of the Mg^2+^ concentration (Figure 2C and D). While the RISC maturation of the siRNA was considerably slower than that of the miRNA at 0.75 mM Mg^2+^, it was significantly promoted by passenger-strand cleavage in the presence of 3.75 mM Mg^2+^, whereas the PS duplex was minimally affected (Figure 2C and D), showing that Mg^2+^ is a key determinant of the efficiency of slicer-dependent unwinding. Nevertheless, the PS duplex was comparable to that of siRNA duplex at 37°C in the presence of 0.75 mM Mg^2+^, indicating that slicer-independent unwinding appeared to exert a dominant influence at this temperature (Figure 2E). The increased level of Mg^2+^ at 37°C resulted in the earlier saturation of target cleavages, not only for the siRNA duplex, but also for the miRNA and PS duplexes (Figure 2F), indicating that the enhanced cleavages were mostly due to the tighter binding of targets, rather than their improved unwinding. Based on these results, we concluded that although slicer-dependent unwinding accelerates RISC maturation at low temperatures, its contribution is less important at the physiological temperature of humans.

### Slicer-deficient AGOs are capable of unwinding the siRNA duplex in a temperature-dependent manner

**Figure 3.**
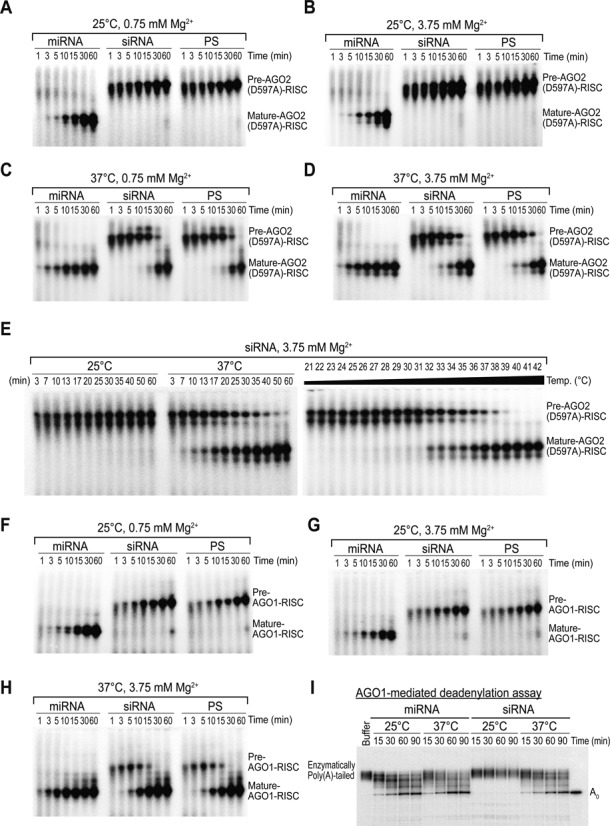
Slicer-deficient AGO proteins can unwind the siRNA duplex in a temperature-dependent manner. (**A**–**D**) PIWI catalytic mutant AGO2 is capable of unwinding the siRNA duplex. Small RNA duplexes containing radiolabeled guide strands were incubated in lysates expressing tagged AGO2 (D597A) at the indicated temperature and Mg^2+^ concentrations for the indicated times. Representative data of at least two independent experiments are shown. (**E**) Higher temperature drives the unwinding of the siRNA duplex when slicer-assisted pathway is not available. Left: Small RNA duplexes containing radiolabeled guide strands were incubated in lysates expressing tagged AGO2 (D597A) at the indicated temperature and Mg^2+^ concentrations for the indicated times. Right panel: 30 min of RISC assembly at the indicated temperature using a temperature gradient in a PCR machine. (**F**–**H**) Non-slicer AGO protein can unwind the siRNA duplex at the physiological temperature of humans. Small RNA duplexes containing radiolabeled guide strands were incubated in lysates expressing tagged AGO1 at the indicated temperature and Mg^2+^ concentrations for the indicated times. Representative data of at least two independent experiments are shown. (**I**) The siRNA duplex is only functionally active at 37°C. Deadenylation by AGO1-RISC was monitored in lysates expressing tagged AGO1 at the indicated temperature and 3.75 mM Mg^2+^ at the indicated times.

Although it has widely been considered that slicer-dependent unwinding via passenger-strand cleavage is required for highly complementary siRNAs, our observations suggest that ‘slicer-dependency’ significantly decreases with increasing temperature. To more directly validate this hypothesis, we monitored RISC assembly using lysates expressing PIWI catalytic mutant AGO2 (D597A) with impaired slicer activity (Supplementary Figure S2B). As expected, only the miRNA achieved full maturation at 25°C, and an increased level of Mg^2+^ did not influence the unwinding of the siRNA, indicating that the slicer-assisted pathway is not available in this mutant (Figure 3A and B). Nonetheless, both the siRNA and PS duplexes were unwound efficiently at 37°C (Figure 3C and D), albeit more slowly than by wild-type AGO2 (Figure 2E and F). Time-course and temperature gradient experiments demonstrated that the higher temperature drives the unwinding of the highly complementary siRNA when slicer-dependent unwinding is not available (Figure 3E).

If the catalytically defective AGO2 mutant is capable of unwinding the siRNAs, is that also the case for other human AGOs (1, 3 and 4) that do not possess slicer-activity? To answer this question, we tested the RISC assembly of other AGOs as well. Similar to the AGO2 catalytic mutant, only miRNA was unwound efficiently at 25°C by AGO1 (Figure 3F), and a higher Mg^2+^ concentration did not accelerate unwinding (Figure 3G). However, RISC maturation was again feasible at 37°C for both the siRNA and PS duplexes (Figure 3H and Supplementary Figure S3A). This conclusion was further supported by an AGO1-mediated deadenylation assay showing that the siRNA is only functionally active at 37°C (Figure 3I). As expected, human AGO3 and 4 exhibited a similar pattern (Supplementary Figure S3B and C), indicating that all four human AGOs have an intrinsic ability to unwind the highly complementary siRNA independently of slicer-activity at their physiological temperature.

### Spontaneous unwinding does not occur before duplexes are loaded into the AGO protein

**Figure 4.**
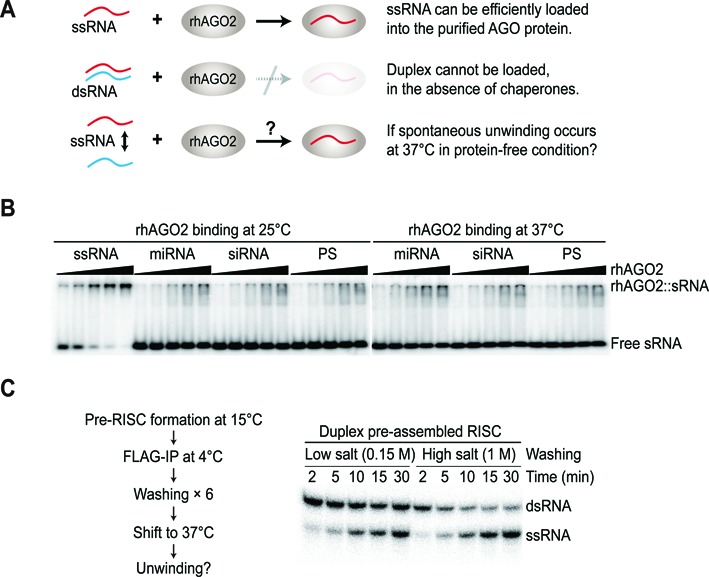
Temperature *per se* is not sufficient to induce duplex unwinding, but requires prior AGO loading. (**A**) A schematic of the experimental design. ssRNA, but not dsRNA, can be incorporated efficiently into purified human AGO2 protein in the absence of chaperone proteins. If the duplex is spontaneously unwound at 37°C, the resulting ssRNA might then be loaded into the purified AGO2 protein. (**B**) None of the duplexes spontaneously unwound at 37°C and form a complex with the purified AGO2 protein. Radiolabeled ssRNA or duplexes (with radiolabeled guide strands) were incubated with increasing concentrations of recombinant hAGO2 for 30 min at the indicated temperatures. Recombinant hAGO2 forms active complexes only with ssRNA. Representative data of at least two independent experiments are shown. (**C**) Prior duplex association with the AGO protein is necessary for duplex unwinding. siRNA duplexes carrying radiolabeled guide strands were incubated with lysates expressing tagged AGO2 for 1 h at 15°C, where duplex loading, but not unwinding, is permissible. Pre-RISCs were subsequently immunopurified at 4°C and washed six times with either low salt (150 mM) or high salt (1 M) before shifting the temperature to 37°C.

We have shown that temperature is the major determinant for RISC maturation, especially when the slicer-assisted pathway is not functional. Considering that duplex stability is affected by temperature, we examined whether spontaneous unwinding occurs before duplexes are loaded into the AGO protein. To test this, we designed an experiment, which is shown in Figure 4A. Canonical RISC loading exclusively requires ATP-dependent chaperone proteins (3–6), whereas a purified, recombinant AGO protein can only incorporate single-stranded RNAs (ssRNAs) via ‘bypass’ loading (11,21,39,40) (Figure 4A). Consistent with the previous observations, our native gel-shift assay demonstrated that purified human AGO2 alone cannot utilize double-stranded RNA (dsRNA) efficiently, and that only ssRNA can be programmed completely (Supplementary Figure S4A–C). If temperature changes alone can drive the unwinding of dsRNA spontaneously, the resulting ssRNA can then be successfully incorporated into the purified hAGO2 protein (Figure 4A). However, none of the small RNA duplexes, even the miRNA with an unstable duplex structure, spontaneously unwound at 37°C and formed a complex with the purified hAGO2 protein (Figure 4B), indicating that temperature change *per se* is not sufficient for duplex unwinding to occur.

To address whether duplex loading is a prerequisite for unwinding, we first assembled pre-RISCs in lysates expressing FLAG-AGO2 at 15°C, where duplex loading, but not unwinding, is permissible. We then immunopurified pre-RISCs containing radiolabeled duplexes, washed them extensively at 4°C, using a high salt (1 M NaCl) buffer to remove ATP and most of the associated proteins, and finally shifted the temperature to 37°C. When the duplex was pre-loaded into the AGO protein, dsRNA was unwound efficiently (Figure 4C), indicating that the association of the duplex with the AGO protein is obligatory for duplex unwinding to occur. Additionally, our results are in agreement with those of previous reports showing that duplex unwinding is a passive process that does not require ATP hydrolysis (2,4,19,21).

### Slicer-independent unwinding depends on the thermodynamic stability of the duplex structure

**Figure 5.**
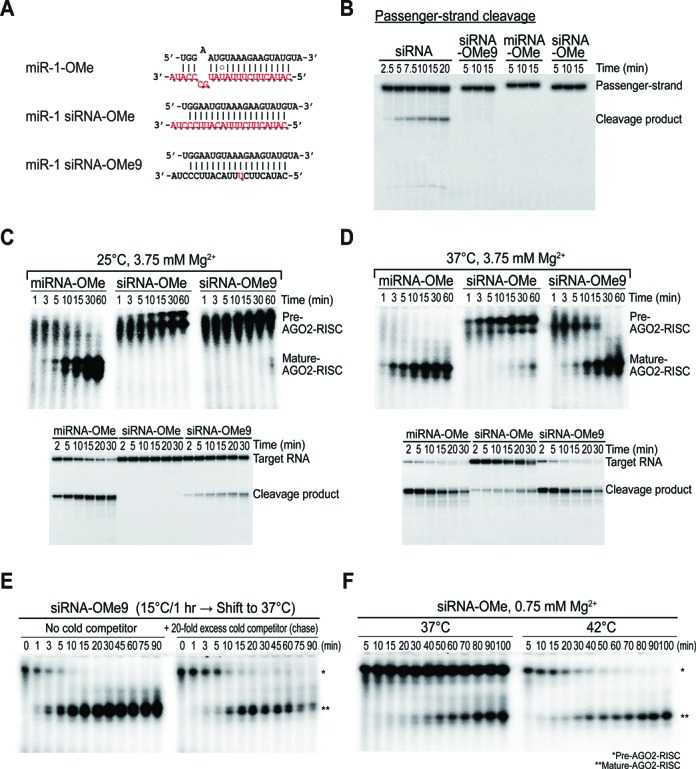
Effects of duplex thermodynamic stability on slicer-independent unwinding. (**A**) 2′-*O*Me modified small RNA duplexes used in this study; miR-1-OMe and miR-1 siRNA-OMe (full 2′-*O*Me-modified passenger-strand) and miR-1 siRNA-OMe9 (with 2′-*O*Me modification only at the ninth of the passenger-strand). Unmodified and 2′*O*Me-modified nts (denoted by *N*_m_) are shown in black and red, respectively. (**B**) 2′-*O*Me-modification completely blocks the cleavage of the passenger-strand. Small RNA duplexes containing radiolabeled passenger-strands were incubated in lysates expressing tagged AGO2 for the indicated times at 25°C in the presence of 5 mM Mg^2+^ to ensure efficient passenger-strand cleavage. Unmodified siRNA served as a positive control. (**C** and **D**) The degree of RISC maturation at different temperatures depends on duplex stability. Small RNA duplexes containing radiolabeled guide strands were incubated in lysates expressing tagged AGO2 at the indicated temperature and Mg^2+^ concentrations for the indicated times. Under the same conditions, target cleavages were analyzed after 30 min of small RNA assembly. Representative data of at least two independent experiments are shown. (**E**) Slicer-independent unwinding is strictly temperature-dependent. Radiolabeled slicer-resistant duplex (siRNA–OMe9) was first incubated at 15°C for 1 h to assemble the pre-RISCs. Subsequently, a 20-fold excess of non-radiolabeled siRNA-OMe9 duplex (cold-competitor) was added to prevent further incorporation of the radiolabeled duplex (at 0 min) before shifting the temperature to 37°C. (**F**) RISC maturation is closely correlated with parameters that affect duplex stability. RISC assembly was monitored for the highly stable duplex (siRNA–OMe) in lysates expressing tagged AGO2 at the indicated temperature and Mg^2+^ concentrations at the indicated times.

To gain additional mechanistic insights into duplex unwinding, we designed duplexes bearing 2′-*O*-methylated nucleotides (nts) in the passenger-strand. It should be noted that 2′-*O*-methylated nts are not only resistant to nucleases, but also form far more stable duplexes with RNA (41). The miRNA that retained its structure, but which had a fully 2′-*O*-methylated passenger-strand was abbreviated as miRNA-OMe (likewise, the labeled siRNA was abbreviated as siRNA-OMe) (Figure 5A). The siRNA-OMe9 was 2′-*O*-methylated only at the ninth nt from the 5′ end of the passenger-strand (Figure 5A). Unlike the PS modification that includes cleavable chiral isoforms (PO/PS) (10), 2′-*O*-methylation at the ninth nt completely abrogated cleavage, as noted previously (11,12) (Figure 5B).

Similar to the PS duplex, the siRNA–OMe9 duplex was hardly unwound at 25°C, but efficiently unwound at 37°C, confirming that passenger-strand cleavage *per se* is not required at the physiological temperature of humans (Figure 5C and D and Supplementary Figure S5A). To more directly test whether slicer-independent unwinding is strictly dependent on temperature, we formed pre-RISCs at 15°C for 1 h, added a 20-fold excess of cold siRNA-OMe9 duplexes (at 0 min) to deter further incorporation of radiolabeled duplexes into pre-RISCs, and shifted the temperature from 15°C to 37°C to monitor the ‘pulse-chase’ conversion of RISC maturation. Pre-RISCs were chased efficiently into mature RISCs (Figure 5E), showing that temperature is the principal controlling factor for slicer-independent unwinding. To our surprise, the miRNA-OMe was fully matured and functionally active at both 25°C and 37°C (Figure 5C and D), indicating that nuclease-mediated degradation of the passenger-strand was not obligatory, at least for an inherently unstable duplex. While duplex unwinding of the siRNA-OMe was significantly reduced (Figure 5C and D), lowering the Mg^2+^ concentration slightly increased slicer-independent unwinding at 37°C (Figure 5F), which may have resulted from a decrease in duplex stability. Complete RISC maturation was achieved at 42°C, although the amount of the RISC complexes decreased, possibly due to the reduced stability of the AGO proteins (Figure 5F). Taken together, we concluded that duplex unwinding and subsequent RISC maturation are highly correlated with certain parameters, such as temperature and Mg^2+^, which affect the stability of the duplex structure.

### Slicer independent unwinding requires the functional domains of the AGO protein

**Figure 6.**
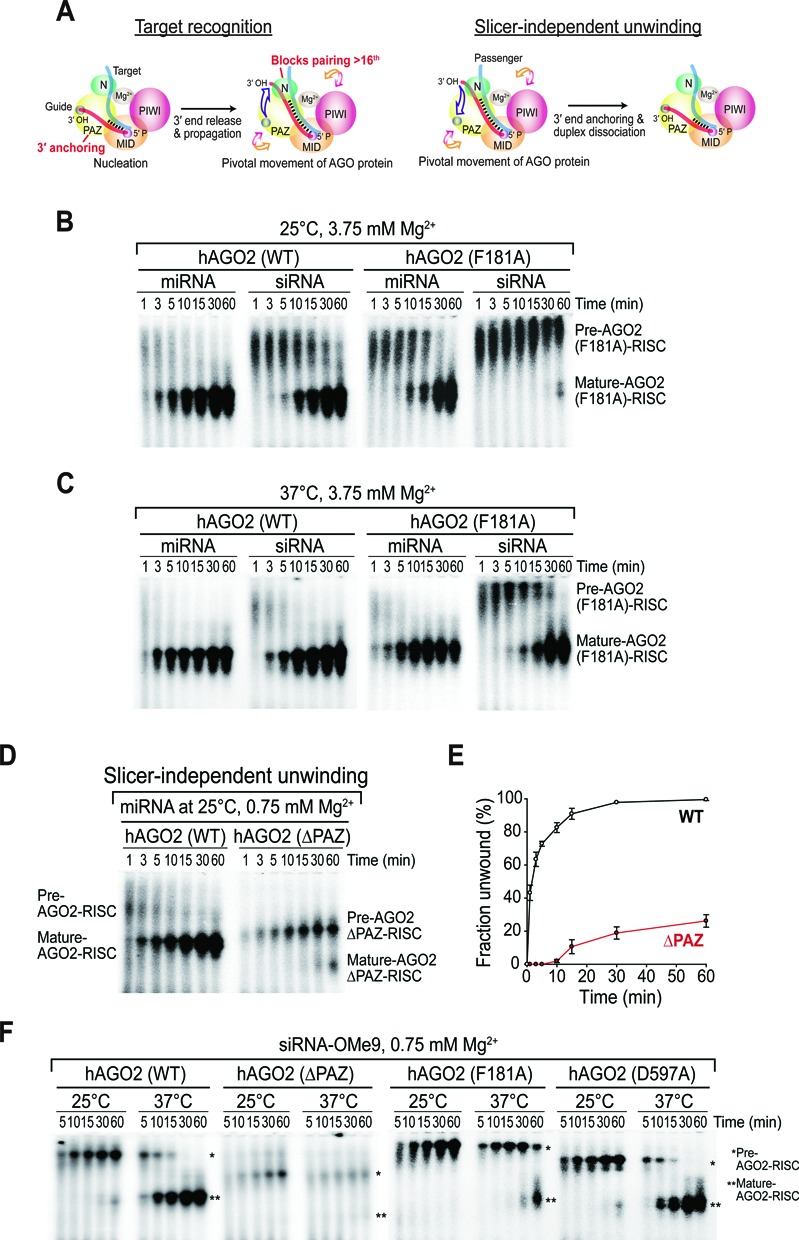
Slicer-independent unwinding is mediated by the functional domains of the AGO protein. (**A**) Left panel: A structural representation of the AGO protein during target RNA recognition. The guide strand (red) binds to its target (light blue) from the 5′ seed-portion (nucleation) and extends to form a double helix (propagation) that terminates at position 16, which is blocked by the N domain. During the propagation step, the 3′ end of the guide is released from its anchor site in the PAZ domain with the help of pivotal movement of AGO. Right panel: Slicer-independent unwinding is assumed to be the reverse process of target RNA recognition, with the passenger-strand being a target. The N and PAZ domains might play a role in duplex unwinding because the N domain blocks additional pairing (beyond 16th) at the 3′ end, which could be further disrupted by dynamic 3′ anchoring of the PAZ domain. (**B** and **C**) N mutant (F181A) is defective for both slicer-dependent and -independent unwinding. Small RNA duplexes containing radiolabeled guide strands were incubated in lysates expressing tagged wild-type and N mutant (F181A) AGO2 proteins at the indicated temperature and Mg^2+^ concentrations for the indicated times. A higher temperature could partially compensate for the wedging defect. Representative data of at least two independent experiments are shown. (**D**) PAZ truncation causes a severe defect in slicer-independent unwinding. miRNA duplexes containing radiolabeled guide strands were incubated in lysates expressing tagged wild-type or PAZ-truncated AGO2 proteins at the indicated temperature and Mg^2+^ for the indicated times. Note that the size of the ΔPAZ-RISC complexes shifted downward due to the truncation of the PAZ domain. (**E**) Quantitation of (D). Data are mean ± SD for two independent experiments. (**F**) Functional domains of AGO2 are required for unwinding, but AGO2-mediated slicer-activity *per se* is dispensable at the physiological temperature of humans. Slicer-resistant duplex (siRNA–OMe9) containing radiolabeled guide strand was incubated in lysates expressing tagged wild-type, PAZ truncation (ΔPAZ), N mutant (F181A) and PIWI catalytic mutant (D597A) AGO2 proteins at the indicated temperature and Mg^2+^ concentration for the indicated times.

As previously proposed, slicer-independent unwinding is the reverse process of target recognition, with the passenger-strand being a target (2,18,19) (Figure 6A). Structural studies have shown that during target recognition (nucleation → propagation), the N domain restricts additional base-pairing (beyond 16th) and the PAZ domain dynamically interacts with the 3′ end of the guide strand, with the help of the pivotal movement of the AGO proteins (24) (Figure 6A). Such ‘wedging’ of the N domain and/or 3′ end anchoring of the PAZ domain are expected to antagonize base-pairing, and thereby pry the duplex apart, as previously reported (18,22).

Given that temperature severely influences the efficiency of slicer-independent unwinding, we further examined the effects of temperature, with consideration of these functional domains. We initially tested an N domain mutant (F181A) AGO2 (Supplementary Figure S5B) that is defective for unwinding, but not for target cleavage (22). At 25°C, the siRNA duplex was unwound efficiently via passenger-strand cleavage in the presence of 3.75 mM Mg^2+^ by wild-type AGO2, whereas the N domain mutant failed to generate a mature RISC under the same conditions (Figure 6B), suggesting that ‘wedging’ is required mostly at the early stage of RISC assembly prior to the cleavage of the passenger-strand. This strong defect of the N domain mutant was partly rescued for the miRNA duplex, although unwinding was still less efficient than for wild-type AGO2 (Figure 6B). Interestingly, the residual defects were largely rescued at 37°C, indicating that higher temperatures could partially offset the wedging defects (Figure 6C).

We further investigated the effect of more severe mutations by creating truncated AGO2 proteins that completely lacked the PAZ or N domains (ΔPAZ or ΔN, respectively) (Supplementary Figure S5C). Although the ΔN protein was not competent to form RISC complexes (Supplementary Figure S5D), the ΔPAZ protein was able to load miRNA duplexes, as previously shown in an *in vivo* study (18) (Figure 6D). For wild-type AGO2, the miRNA duplex was unwound almost instantaneously, whereas the ΔPAZ protein exhibited a severe defect (Figure 6D and E), indicating that a functional PAZ domain is strictly required for slicer-independent unwinding. When tested with the slicer-resistant siRNA (OMe9), the ΔPAZ protein could not load the siRNA duplex effectively (Figure 6F). A less severe mutant (F181A) could form the pre-RISC successfully, but exhibited much slower unwinding activity than wild-type AGO2, whereas a PIWI catalytic mutant (D597A) displayed no defects (Figure 6F). Taken together, we conclude that functional N and PAZ domains are required for slicer-independent unwinding, whereas slicer-activity of AGO2 *per se* is largely dispensable at the physiological temperature of humans.

### Slicer-independent unwinding is a general mechanism for human RISC maturation

Our data strongly suggest that slicer-independent unwinding is likely to play a dominant role in human RISC. To substantiate this idea *in vivo*, we designed ‘artificial’ siRNA duplexes targeting GAPDH mRNA (siGAPDH) (Supplementary Figure S6A). The ninth nt from the 5′ end of the passenger-strand was modified by a 2′-*O*-methyl group (siGAPDH-OMe9) that completely blocked passenger-strand cleavage (Supplementary Figure S6B). While the knockdown effects were not appreciable at 28°C, the siGAPDH–OMe9 duplex displayed a silencing potency comparable to its conventional counterpart under normal cell culture conditions (Supplementary Figure S6C). These results indicate that slicer-independent unwinding plays a general role in human RISC maturation, as was also confirmed in other tested duplexes (Supplementary Figure S6D and E) and via endogenous AGO2 in HeLa cell lysates (Supplementary Figure S6F).

### Slicer-independent unwinding is a conserved mechanism

**Figure 7.**
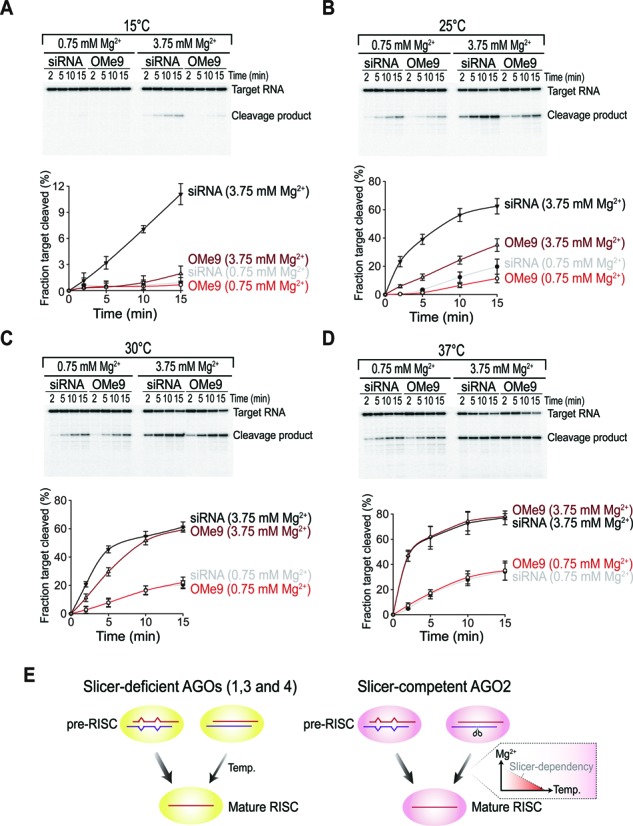
Slicer-independent unwinding is a conserved mechanism. (**A**–**D**) Distinct slicer-dependency at different temperatures in *Drosophila* AGO2. Small RNAs were assembled in S2 cell lysates only for 5 min (to avoid early saturation) at the indicated temperature and Mg^2+^ level. Cap-radiolabeled target RNA was then added and incubated for the indicated times at the indicated temperatures and Mg^2+^ concentrations. Quantitation of the fraction target cleaved (%) is shown below the gel image. Black: siRNA with 3.75 mM Mg^2+^, Brown: siRNA-OMe9 with 3.75 mM Mg^2+^, Gray: siRNA with 0.75 mM Mg^2+^, Red: siRNA-OMe9 with 0.75 mM Mg^2+^. Data are mean ± SD for at least two independent experiments. (**E**) A propose model for small RNA maturation in human RISCs. miRNAs are unwound most efficiently in all four human AGOs through the aid of internal mismatches. Slicer-deficient AGOs (1, 3 and 4) are able to unwind the highly complementary siRNA in a temperature-dependent manner. AGO2 can additionally utilize its slicer-activity for the highly complementary siRNA with slicer-dependency being positively correlated to the Mg^2+^ concentration and negatively correlated to temperature.

Small RNAs have been documented in varied organisms, and our results raise an interesting question of how temperature affects the RISC maturation of organisms that maintain a wide range of physiological temperatures. *Drosophila* was the first model organism used to study RNAi *in vitro* (28), and its mechanism of action is one of the best characterized. Likewise, slicer-dependent unwinding was mainly demonstrated in *Drosophila* (10,11,13), which shows a temperature preference for 25°C. Is the mechanism of slicer-independent unwinding also conserved in *Drosophila*? To answer this question, we performed a fly AGO2-mediated target cleavage assay using S2 cell lysates at various ambient temperatures. Only the siRNA exhibited a minimal cleavage activity at 15°C in the presence of 3.75 mM Mg^2+^, indicating that slicer-independent unwinding was not available at this temperature (Figure 7A). At the physiological temperature of *Drosophila*, the siRNA produced active RISCs, whereas the OMe9 duplex did much less efficiently (Figure 7B), suggesting that passenger-strand cleavage greatly accelerates the rate of RISC assembly at 25°C, as shown previously (10,11,13). In contrast, the contribution of the slicer-dependent pathway became much less important when the temperature was increased to 30°C (Figure 7C). Finally, the slicer-independent pathway appeared to exert a predominant influence on RISC maturation at 37°C, the physiological temperature of humans (Figure 7D). An extended incubation (>30 min) at 37°C severely impaired RISC activity (data not shown), indicating that the *Drosophila* AGO protein does not function optimally (and is even possibly detrimental) at high temperature. Based on these results, we concluded that slicer-independent unwinding is likely to be a conserved mechanism for RISC maturation, although the extent to which the slicer-dependent pathway contributes to RISC maturation may vary greatly, depending on the respective body temperatures of living organisms.

## DISCUSSION

Since the first discovery of the passenger-strand cleavage mechanism a decade ago, it has been widely recognized that slicer-dependent unwinding is a prerequisite for the assembly of highly complementary siRNAs into RISCs (10,11,13). Our collective results indicate that this assumption is typically valid, but not always, particularly for mammals and birds. Earlier biochemical studies relied mainly on target cleavage assays to understand the overall nature of RISC catalysis, which often tended to dismiss important differences during the early stages of RISC assembly. Through a careful re-examination of RISC assembly using a variety of several biochemical analyses, including duplex loading, slicer-dependent and -independent unwinding, and classical target cleavage assays, we established here that slicer-independent unwinding is a more prevalent mechanism for human RISC maturation than previously thought, not only for miRNA duplexes but also for highly complementary siRNAs as well. Aside from the main findings, there are several other findings that are important to discuss in detail.

### Small RNA sorting and the Mg^2+^ level in slicer-dependent unwinding

It has been previously recognized that only AGO2 can efficiently unwind siRNA duplexes via passenger-strand cleavage in humans (21), and this is reasonable in the sense that AGO2 is among the most advantageous human AGO proteins that can utilize its slicer-activity (42,43) for RISC maturation (Figure 7E). Nonetheless, we showed that cleavage-deficient AGOs (1, 3, and 4) can also be programmed with siRNAs at the physiologically relevant temperature of humans, suggesting that slicer-independent unwinding is likely a common feature of human AGO proteins. These observations provided a natural explanation for why both miRNAs and siRNAs are found in all four human AGO proteins, irrespective of their sequences (44,45). In contrast with weak small RNA sorting in humans, siRNA duplexes are specifically sorted into fly AGO2 (46,47), which is essential for antiviral defense (48). The fly AGO2 should therefore acquire an additional strategy for siRNA maturation (i.e., passenger-strand cleavage), otherwise not efficient at their body temperature.

We showed that a certain level of Mg^2+^ is required for slicer-dependent unwinding to occur efficiently (Figure 2A). While the free cytosolic Mg^2+^ level is less than 1 mM under normal conditions (49), the total cellular Mg^2+^ concentration can vary from 5 to 20 mM (50,51), as most Mg^2+^ ions are bound to proteins and negatively charged molecules (50,51). Therefore, it is difficult to estimate the exact amount of Mg^2+^ bound to AGO2, although previous studies typically used 1.5–5 mM Mg^2+^ for the *in vitro* slicing assays (10,11,42,43,52). In addition, cytosolic Mg^2+^ levels can be altered by ATP, which is capable of chelating Mg^2+^ ions (53). In other words, a transient decrease in ATP levels may give rise to a sudden increase in the Mg^2+^ concentration (53). During two steps of RISC assembly, duplex loading requires ATP hydrolysis, and subsequent cleavage of the passenger-strand requires a relatively high level of Mg^2+^. It is tempting to speculate that bursts of metabolic activity during pre-RISC formation may induce a transient decrease in ATP that results in an increase in Mg^2+^ levels that allows for more efficient slicer-activity, although it is technically difficult to demonstrate such rapid and transient changes.

### Slicer-independent unwinding in human RISCs

Although chaperone machinery-mediated duplex loading is relatively well understood (3–6), it is still remain elusive how the loaded duplex unwinds to form the mature RISC. The helicase model was proposed at the beginning of the RNAi field — an ATP-dependent helicase separates the two strands of the duplex before they are loaded into the AGO protein (30), although such an ‘unwindase’ has yet to be identified (2). Another model assumes that duplexes are loaded into the AGO protein, which itself can dissociate the two strands (2,18,19,22). Our findings, combined with those of previous studies, point to the latter model as a more plausible scenario. It has been extensively demonstrated that AGO proteins receive duplexes, rather than single strands, during RISC assembly (4,5,10–13,19,21). Once a duplex is deeply buried within the AGO protein, it is difficult to comprehend how the duplex is further transferred from AGO proteins to other regulatory factors in an accessible form. We showed that the unwinding process was significantly influenced by intrinsic factors, such as temperature and Mg^2+^, both of which were closely related to duplex stability. Our functional analyses also indicated that the AGO protein itself is a key factor that is needed for duplex unwinding. Recent structural studies have corroborated the idea that target dissociation is strongly coupled to a profound conformational change in the AGO proteins, which results in a widening of the N-PAZ channel, thereby leading to a disruption of the base-pairing in the 3′ half of the guide (54,55). We postulate that high temperature may favor a conformational change (56,57) in the AGO protein that accelerates RISC maturation.

miRNAs are the most abundant and common endogenous substrates for human AGOs and they often have multiple mismatches and G-U wobble base pairs (or even bulges) that enable highly efficient unwinding (Figure 7E and Supplementary Figure S6E). In contrast, a highly complementary siRNA duplex requires either a certain temperature or AGO2-mediated cleavage of passenger-strands, which results in a drastic change in the thermodynamic profile of the duplex. siRNA unwinding by AGO2 depends upon two main factors with opposite effects: temperature and Mg^2+^ (Figure 7E). At the physiological temperature of humans, siRNAs could also be incorporated into slicer-deficient AGOs (1, 3 and 4) to form the mature RISC (Figure 7E). In mammals, endogenous siRNAs can repress complementary mRNAs and transposons in mouse oocytes (58) and embryonic stem cells (59), although little is known about their biological role.

### A thermodynamic perspective of RISC maturation

In light of our current findings, as well as those of previous reports, we envision that the overall process of RISC assembly has characteristics closely resembling those of a classical thermodynamic reaction (Supplementary Figure S7). Duplex loading requires the energy from ATP hydrolysis (i.e. *E*_a_) to trigger a conformational change in the AGO proteins that is mediated by the Hsc70–Hsp90 chaperone machinery (3–6). In this regards, the pre-RISC can be considered to be a transition state during RISC assembly (Supplementary Figure S7). The mature RISC is thought to be the most stable and energetically favorable state (Δ*H* < 0) (Supplementary Figure S7). This assumption is supported by the fact that AGO proteins mostly co-purify with endogenous guide RNAs and were stable enough to be structurally resolved by crystallography (33,34,54,60).

Because duplex unwinding is generally accompanied by an increase in entropy (61,62) (Δ*S* > 0), the spontaneity (Δ*G* = Δ*H* − *T*Δ*S*) of RISC maturation is then largely influenced by the temperature that contributes to the entropy of the system. Although this may seem to be plausible, we cannot warrant further speculation with our present biochemical data alone. Future studies combining structural analyses with biophysical techniques are necessary to reveal the structural basis for duplex unwinding, as well as its thermodynamics.

Homeotherms, such as mammals and birds, have a specific physiological adaptation that enables them to regulate their body temperature from 36 to 42°C (63). In contrast, poikilotherms, including worms, flies, plants, and fish, lack the means to generate heat (64). Therefore, the body temperatures of these animals tend to conform to their external environment, and temperature fluctuations may affect numerous aspects of their physiology, including enzyme function, muscle activity, and energy metabolism (64). Our results suggested that the last step of small RNA biogenesis could be largely influenced by such temperature changes, which may provide a means to fine-tune the expression of small RNAs in various living organisms.

## Supplementary Material

SUPPLEMENTARY DATA
