# Supplementary material for: Slicer-independent mechanism drives small-RNA strand separation during human RISC assembly
Source: Nucleic Acids Res. 2015 Sep 17;43(19):9418–33. doi: 10.1093/nar/gkv937 (PMC4627090; doi:10.1093/nar/gkv937)
Supplement: SUPPLEMENTARY DATA [file supp_43_19_9418__index.html]

Slicer-independent mechanism drives small-RNA strand separation during human RISC assembly — Slicer-independent mechanism drives small-RNA strand separation during human RISC assembly — SUPPLEMENTARY DATA 

# Slicer-independent mechanism drives small-RNA strand separation during human RISC assembly

## SUPPLEMENTARY DATA

- SUPPLEMENTARY DATA
