# Supplementary material for: Slicer-independent mechanism drives small-RNA strand separation during human RISC assembly
Source: Nucleic Acids Res. 2015 Sep 17;43(19):9418–33. doi: 10.1093/nar/gkv937 (PMC4627090; doi:10.1093/nar/gkv937)
Supplement: SUPPLEMENTARY DATA [file supp_gkv937_nar-01923-y-2015-File009.pdf]

Supplementary Figure 1

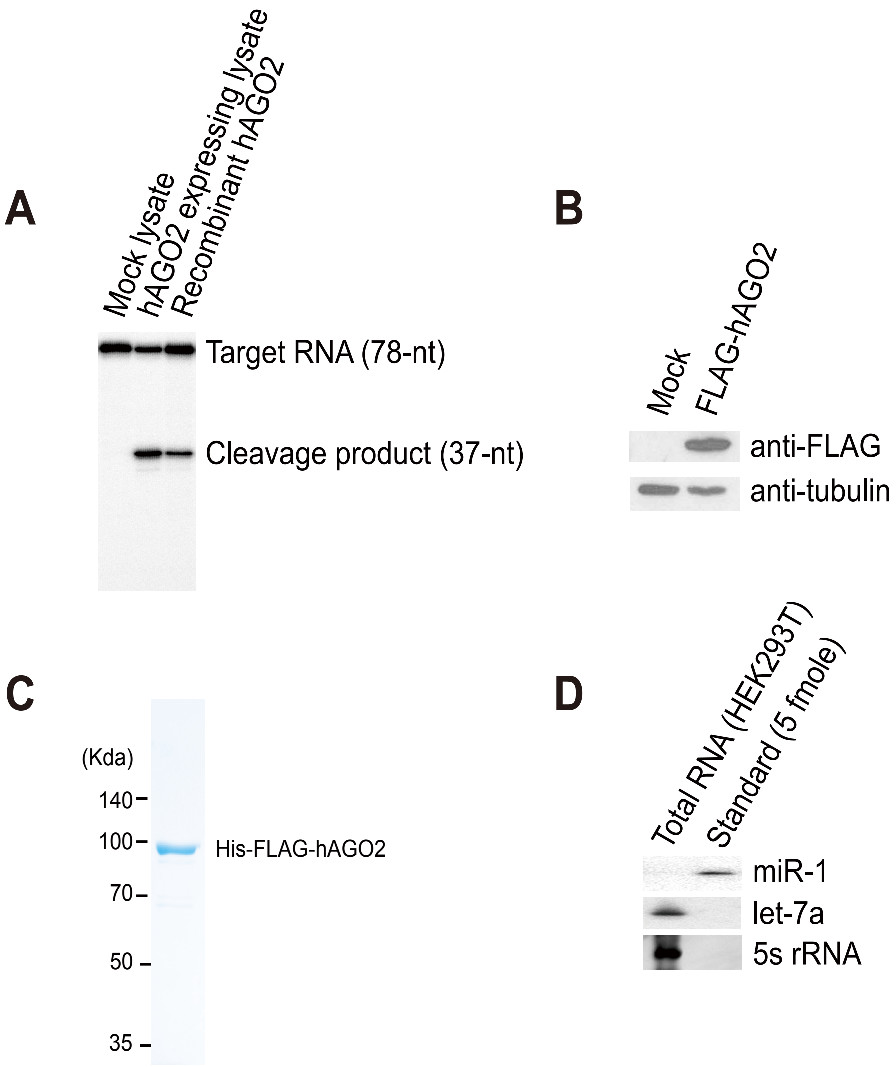

Supplementary Figure 1. Mammalian cell-free system from HEK293T cells that faithfully recapitulates RNAi *in vitro.* (A) miRNA duplex was assembled in lysates from naive HEK293T cells (Mock) and from cells expressing FLAG-hAGO2 for 15 min. Fifty nanomolar of recombinant hAGO2 protein was programmed with a single-stranded guide RNA. Cap-radiolabeled target RNA (78-nt) was then added and further incubated for 15 min, which yielded a 5′ cleavage product at the identical position (37-nt), as a diagnostic for hAGO2-mediated catalysis. (B) Western blot analysis using an anti-FLAG antibody confirmed the expression of tagged AGO2 protein. Anti-tubulin served as an internal control. (C) Coomassie-staining of tandem affinity purified recombinant human AGO2 protein derived from the baculovirus system. (D) Thirty micrograms of total RNA from HEK293T cells was subjected to Northern blot analysis with 5 fmole of synthetic miR-1 standard as a positive control. let-7a and 5s rRNA served as a positive (for total RNA sample) and negative (for synthetic RNA sample) control.

Supplementary Figure 2

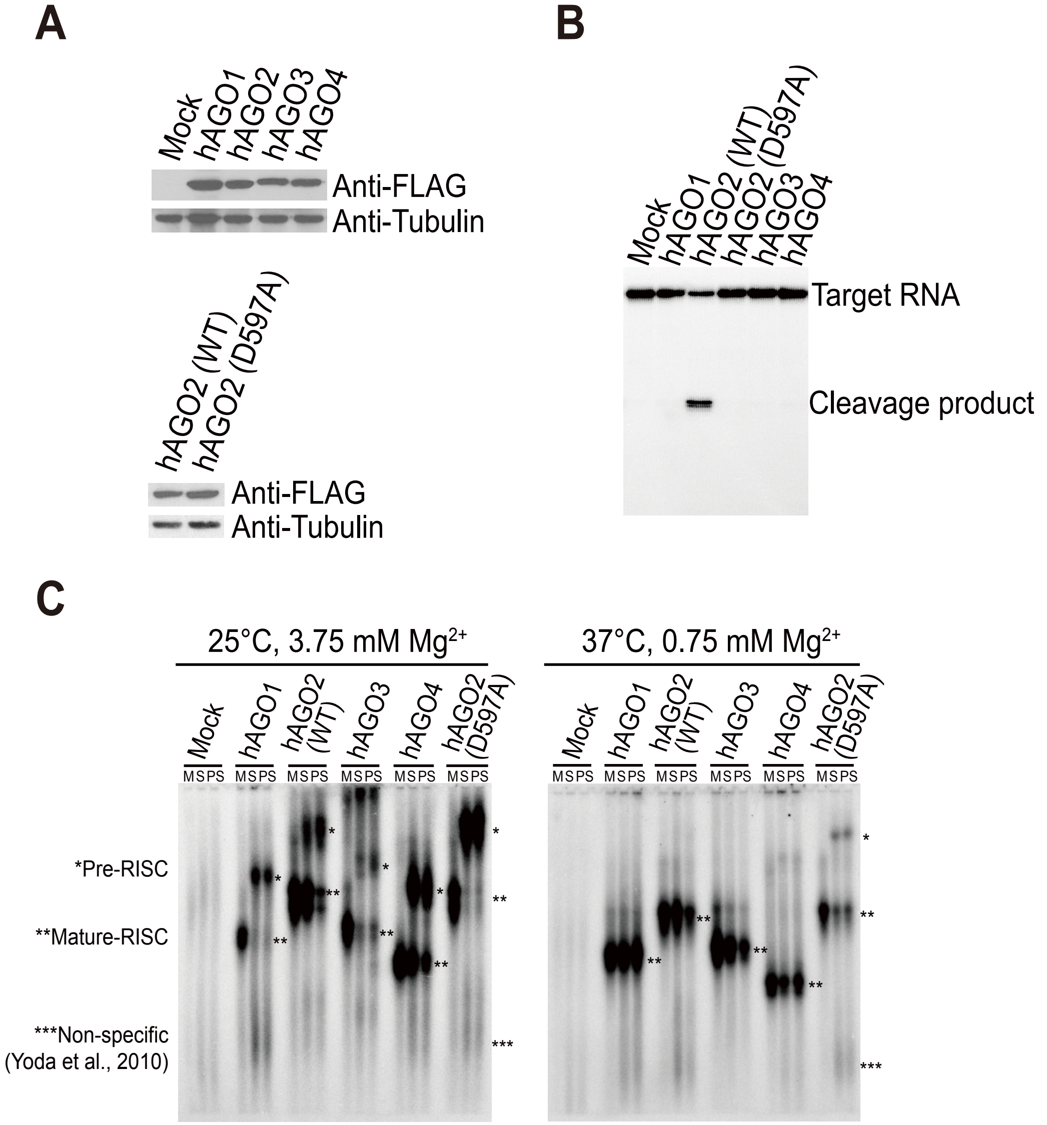

Supplementary Figure 2. Characterization of the RISC complexes detected in lysates from HEK293T cells expressing FLAG-tagged AGO proteins. (A) Western blot analysis using an anti-FLAG antibody confirmed the expression of tagged AGO proteins. Anti-tubulin served as an internal control. (B) Target cleavage assay in lysates from HEK293T cells expressing tagged AGO proteins. miRNA duplex was assembled in lysates from naive HEK-293T cells (Mock) and from cells expressing tagged AGO proteins for 15 min. Cap-radiolabeled target RNA was then added and further incubated for 15 min. (C) Small RNA duplexes containing radiolabeled guide strands were incubated in lysates expressing tagged AGO proteins at the indicated temperature and Mg^{2+} for 30 min. The RISC complexes were separated on a vertical agarose native gel at 4°C. The migration of the RISC complexes varied slightly among AGO proteins, possibly due to their different sizes and charges of each AGO protein (Yoda et al., 2010). M; miRNA, S; siRNA, PS; siRNA with phosphorothioate modification. Please refer to main figure 2 and 3 to monitor the overall kinetic behavior of the RISC complexes and dependence on temperature and Mg^{2+} level for each different small RNA duplex.

Supplementary Figure 3

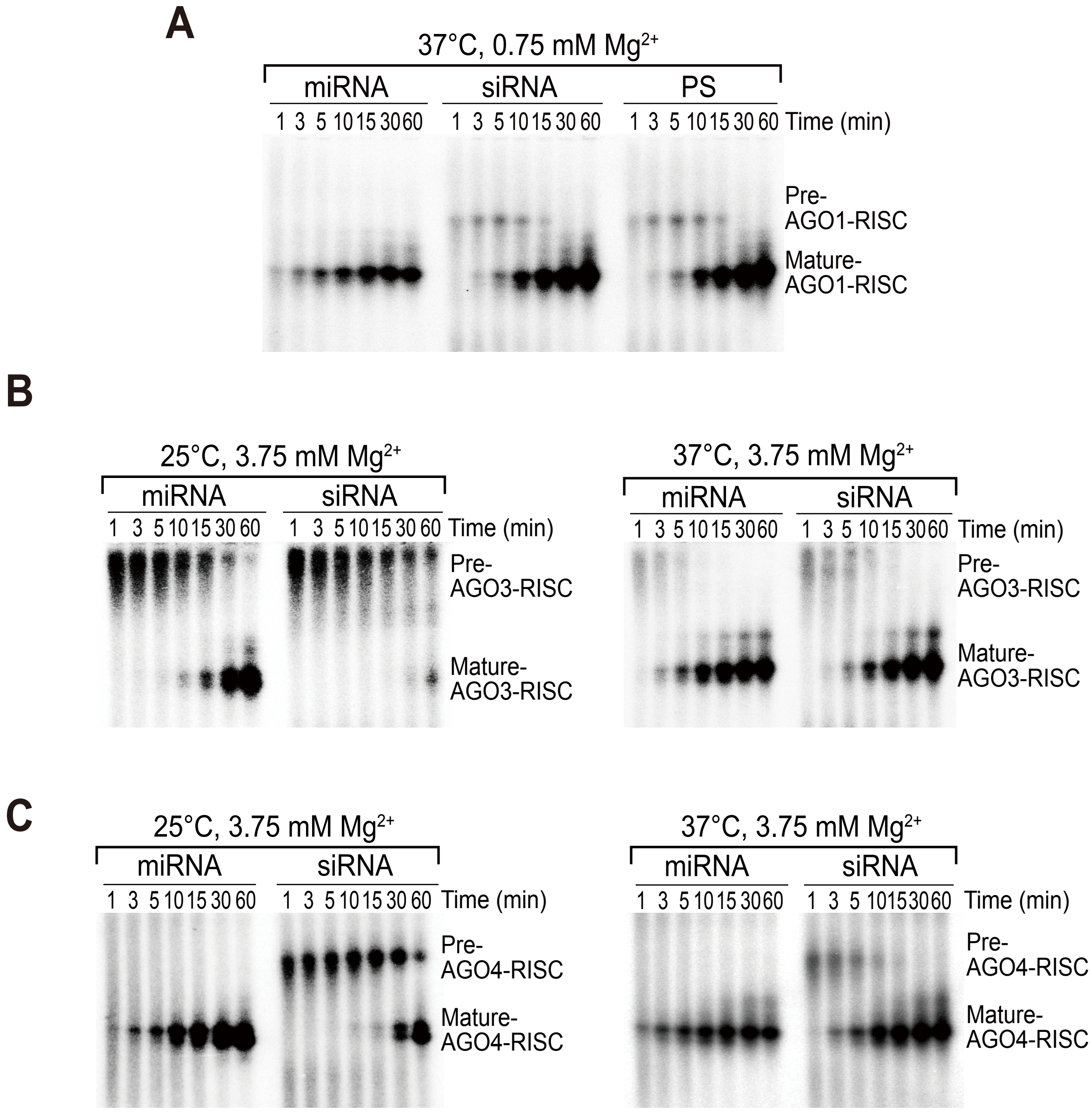

Supplementary Figure 3. Slicer-deficient AGO proteins can unwind siRNA duplex in a temperature-dependent manner. (A-C) Non-slicer AGO proteins are capable of unwinding siRNA duplex at the physiological temperature in human. Small RNA duplexes containing radiolabeled guide strands were incubated in lysates expressing tagged AGO1, AGO3 and AGO4 at the indicated temperature and Mg^{2+} for the indicated time points. The RISC complexes were separated on a vertical agarose native gel at 4°C.

Supplementary Figure 4

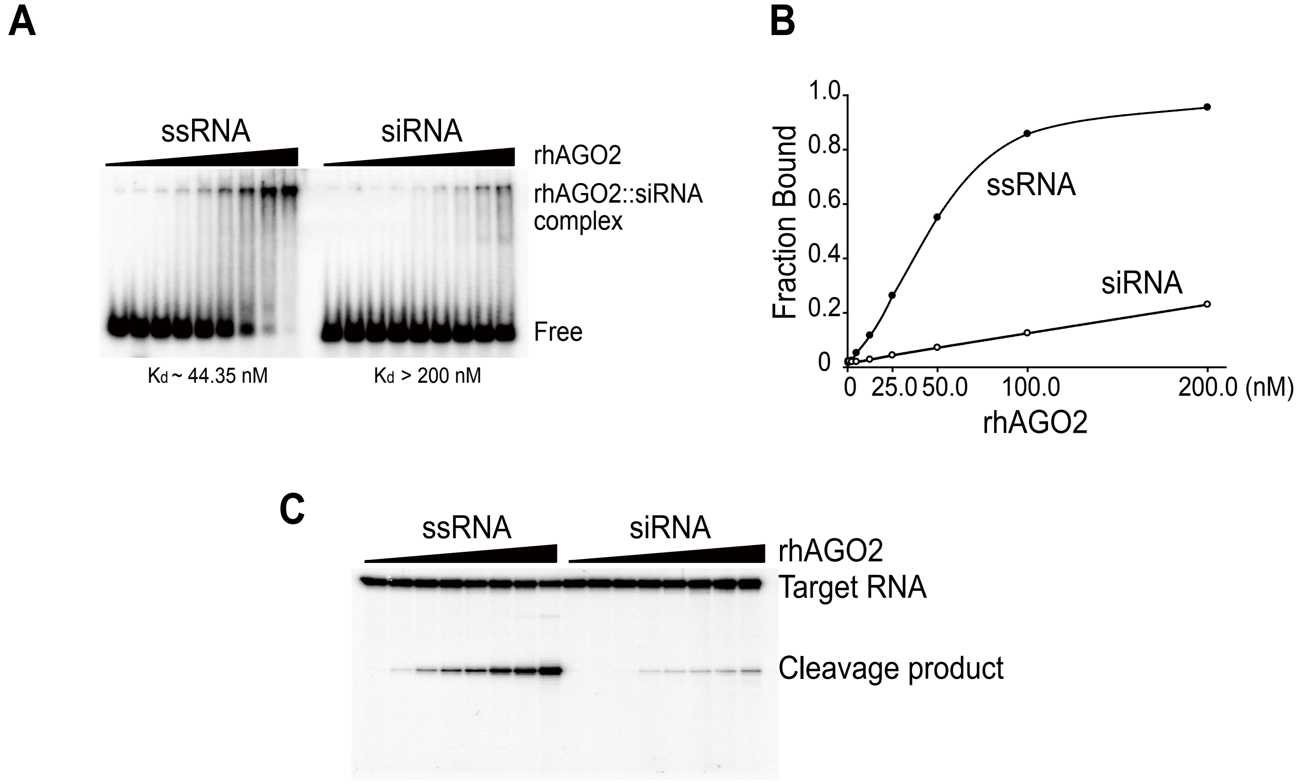

Supplementary Figure 4. Recombinant human AGO2 alone can hardly utilize the siRNA duplex. (A) Radiolabeled ssRNA or siRNA (radiolabeled with guide strand) were incubated with increasing concentration of the recombinant hAGO2 (rhAGO2) for 30 min at 25°C. Complexes were analyzed by 6% native gel electrophoresis at 4°C. Recombinant AGO2 forms active complexes only with ssRNA. (B) The fraction bound to rhAGO2 was plotted against the concentration of the purified proteins. (C) The ssRNA or siRNA were incubated with increasing concentration of the recombinant hAGO2 for 15 min. Cap-radiolabeled target RNA was then added and further incubated for 15 min. The siRNA function much less efficiently than ssRNA in the cleavage assay, consistent with the gel-shift assay in (A).

Supplementary Figure 5

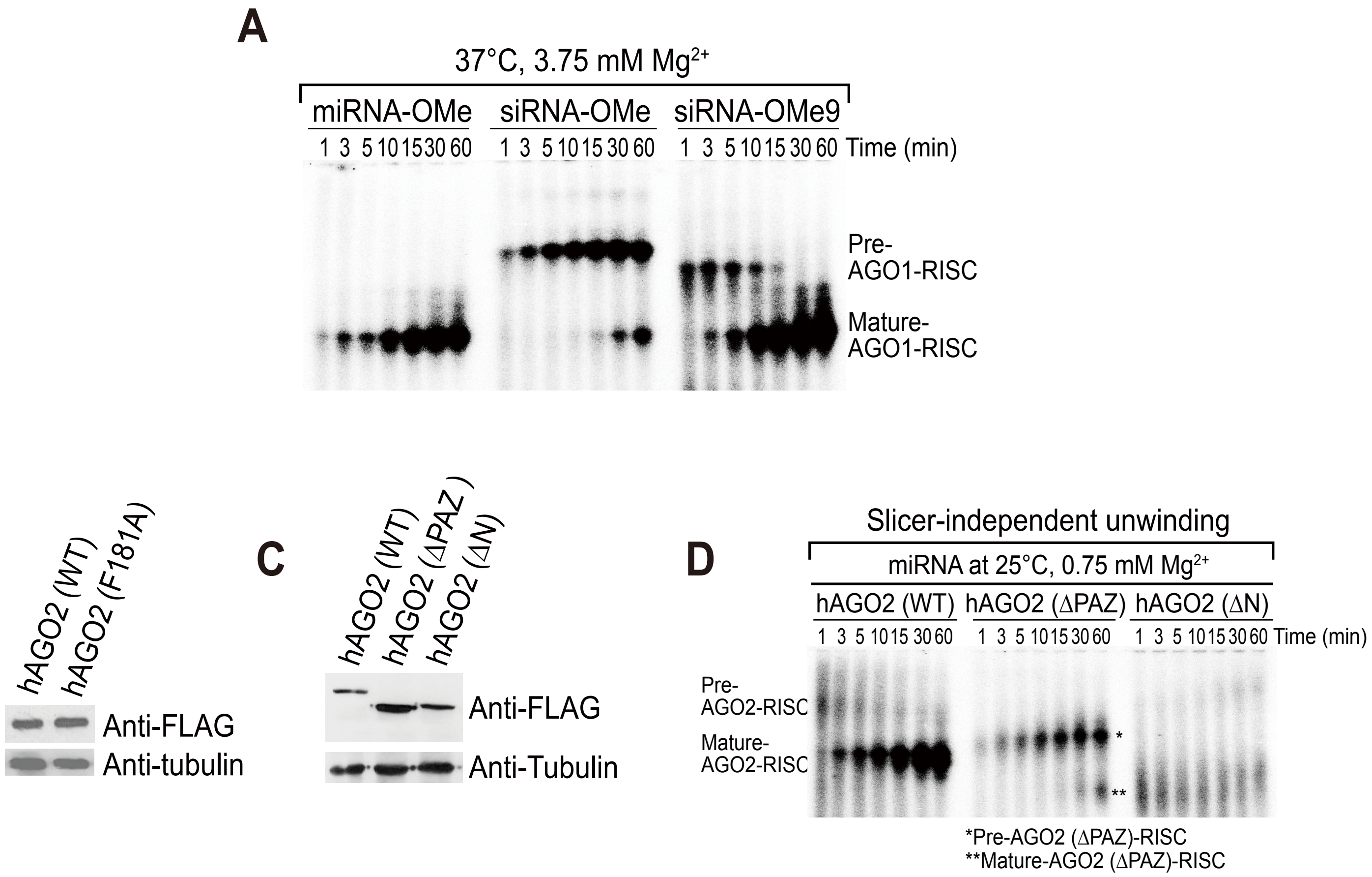

Supplementary Figure 5. Functional domains of the AGO protein are required for slicer-independent unwinding. (A) Effects of duplex stability on slicer-independent unwinding by AGO1 (related to main Figure 5D). Small RNA duplexes containing radiolabeled guide strands were incubated in lysates expressing tagged AGO1 at the indicated temperature and Mg^{2+} concentrations for the indicated times. (B-C) Western blot analysis using an anti-FLAG antibody confirmed the expression of tagged AGO2 mutant proteins. Anti-tubulin served as an internal control. (D) miRNA duplexes containing radiolabeled guide strands were incubated in lysates expressing tagged wild-type and PAZ or N-truncated AGO2 proteins at the indicated temperature and Mg^{2+} for the indicated times.

Supplementary Figure 6

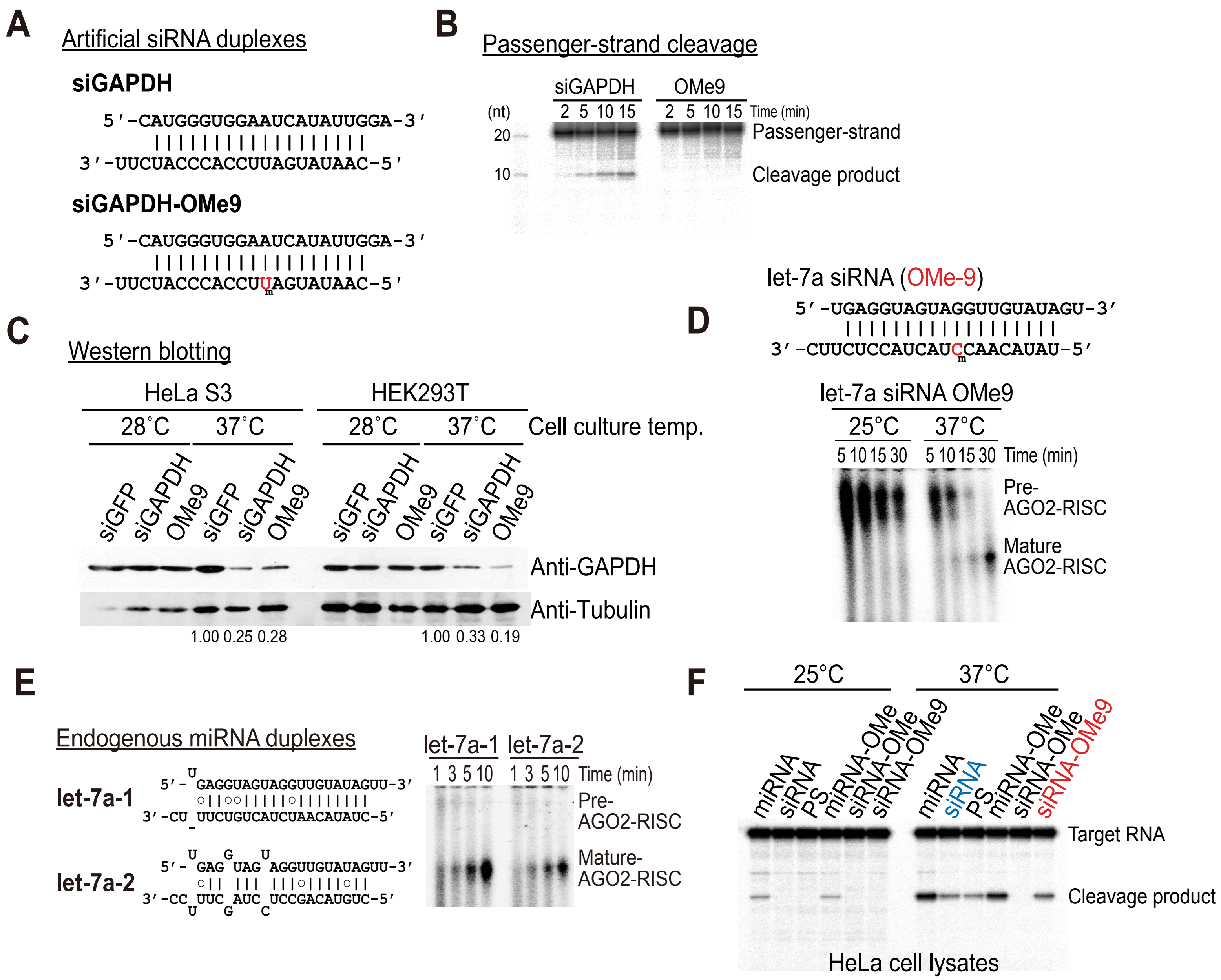

Supplementary Figure 6. Slicer-independent unwinding is a general mechanism for human RISC maturation. (A) Artificially designed siRNA duplexes targeting an endogenous mRNA. siGAPDH; functionally asymmetric siRNA designed to target GAPDH and siGAPDH-OMe9; 2'-OMe modification at the 9th nt to block passenger-strand cleavage. (B) Passenger-strand cleavage in the siGAPDH-OMe9 duplex is abrogated. Small RNA duplexes containing radiolabeled passenger-strands were incubated in lysates expressing tagged AGO2 for the indicated times at 25°C in the presence of 5 mM Mg^{2+} to ensure an efficient passenger-strand cleavage. (C) The siGAPDH-OMe9 duplex can elicit an effective silencing response with similar potency as its conventional counterpart. HeLa S3 cells were transfected with 10 nM of siGFP (mock), siGAPDH and siGAPDH-OMe9. HEK293T cells were co-transfected with 10 nM of the indicated siRNAs and AGO2 plasmids. Cells were then cultured either at 28°C or 37°C and harvested at 24 h post-transfection, and analyzed by western blotting. The numbers below the western blot are the relative expression levels, normalized using β-tubulin loading control. (D) let-7a siRNA OMe9; let-7a was perfectly paired to its antisense, except for the 1st position from the 5' end. 2′-*O*Me modification at the 9th nt from the 5′ end of passenger-strand. Unmodified and 2′-OMe-modified nucleotide (denoted by N_m) are shown in black and red, respectively. let-7a siRNA-OMe9 duplex was hardly unwound at 25°C, but effectively unwound at 37°C, as expected. Small RNA duplex containing radiolabeled guide strand was incubated in lysates expressing tagged AGO2 at the indicated temperature for the indicated times. (E) Endogenous miRNAs often have multiple mismatches and G-U wobble base pairs (let-7a as a representative example) that enable highly efficient unwinding. miRNA duplexes containing radiolabeled guide-strands were incubated in lysates expressing tagged AGO2 at 25°C for the indicated times. miRNA duplexes form mature RISCs rapidly and efficiently. (F) Target cleavage assay in an endogenous AGO2 in HeLa cell lysates. Small RNA duplexes were assembled in naive HeLa cell lysates for 15 min at the indicated temperature before the addition of cap-radiolabeled target RNA. These results, largely consistent with the results from HEK293T cells, suggest that slicer-independent unwinding plays a dominant role in human RISC maturation.

Supplementary Figure 7

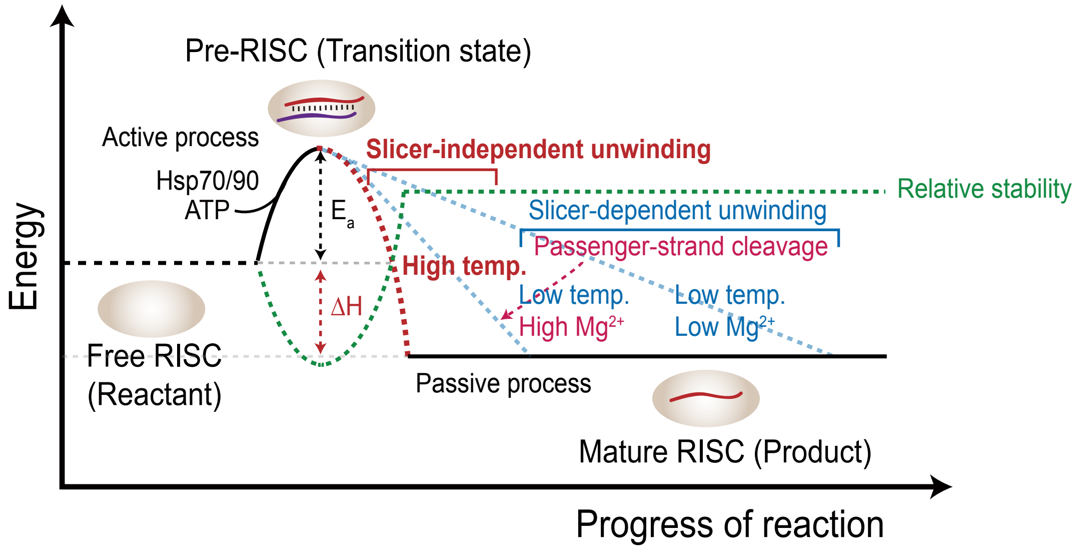

Supplementary Figure 7. An envisioned model for human RISC assembly. An envisioned model is thoroughly described in the discussion section of the subheading 'A thermodynamic perspective of RISC maturation'. We assume that the mature RISC is among the most stable and energetically favorable form, whereas the free- and pre-RISC are expected to be more dynamic. Slicer-independent unwinding (red) plays a dominant role in small RNA maturation at mammalian body temperatures, whereas slicer-dependent unwinding (blue) may serve as an indispensable strategy for organisms with low body temperatures, and it is enhanced by the presence of high Mg^{2+} levels (light red). The relative stability (green) of the RISC complexes is inversely correlated to the energy states. ΔH, enthalpy; E_a, activation energy.

Supplementary Table 1. The sequences of oligonucleotides used for the assays

**Oligonucleotides**	**Sequences (5′-3′)**
miR-1 guide	UGGAAUGUAAAGAAGUAUGUA
miR-1 passenger	CAUACUUCUUUAUAUGCCCAUA
miR-1 siRNA passenger	CAUACUUCUUUACAUUCCCUA
miR-1 siRNA passenger PS	$CAUACUUCU_{(PO/PS)}UUACAUUCCCUA$
miR-1 passenger (2′-OMe)	$C_mA_mU_mA_mC_mU_mU_mC_mU_mU_mU_mA_mU_mA_mU_mG_mC_mC_mC_mA_mU_mA_m$
miR-1 siRNA passenger (2′-OMe)	$C_mA_mU_mA_mC_mU_mU_mC_mU_mU_mU_mA_mC_mA_mU_mU_mC_mC_mC_mU_mA_m$
miR-1 siRNA passenger OMe9	$CAUACUUCU_mUUACAUUCCCUA$
miR-1 guide ASO (2′-OMe)	$U_mC_mU_mU_mC_mU_mA_mC_mA_mU_mA_mC_mU_mU_mC_mU_mU_mU_mA_mC_mA_mU_mU_mC_mC_mA_mA_mC_mC_mU_mU_m$
miR-1 passenger ASO (2′-OMe)	$U_mC_mA_mC_mA_mU_mG_mG_mA_mA_mU_mG_mU_mA_mA_mA_mG_mA_mA_mG_mU_mA_mU_mG_mU_mA_mA_mU_mC_mU_mC_m$
let-7a miRNA guide	UGAGGUAGUAGGUUGUAUAGUU
let-7a siRNA guide	UGAGGUAGUAGGUUGUAUAGU
let-7a-1 passenger	CUAUACAAUCUACUGUCUUUC
let-7a-2 passenger	CUGUACAGCCUCCUAGCUUUCC
let-7a siRNA passenger OMe9	UAUACAACCmUACUACCUCUUC
let-7a guide ASO (2′-OMe)	$U_mC_mU_mU_mC_mA_mC_mU_mA_mU_mA_mC_mA_mA_mC_mC_mU_mA_mC_mU_mA_mC_mC_mU_mC_mA_mA_mC_mC_mU_mU_m$
siGAPDH guide	CAUGGGUGGAAUCAUAUUGGA
siGAPDH passenger	CAAUAUGAUUCCACCCAUCUU
siGAPDH passenger OMe9	$CAAUAUGAU_mUCCACCCAUCUU$
siGAPDH passenger ASO (2′-OMe)	$U_mC_mA_mC_mA_mA_mA_mG_mA_mU_mG_mG_mG_mU_mG_mG_mA_mA_mU_mC_mA_mU_mA_mU_mU_mG_mA_mU_mC_mU_mC_m$
miR-1 antisense DNA probe	TACATACTTCTTTACATTCCA
let-7a antisense DNA probe	AACTATACAACCTACTACCTCA
